# Development of a Novel, Simultaneous Determination Method for Heterocyclic Amines and Mycotoxins in Fried Food by UPLC–MS/MS Combined With QuEChERS

**DOI:** 10.1002/fsn3.71593

**Published:** 2026-04-02

**Authors:** Diaodiao Yang, Jianwen Liu, Yang Song, Yajing Ma, Hongkang Zhu, Qingfeng Zhou, Maomao Zeng

**Affiliations:** ^1^ Henan Engineering Technology Research Center for Functional Development and Application of Characteristic Agricultural Products, Shangqiu Normal University Shangqiu Henan China; ^2^ School of Food Science and Engineering, Yangzhou University Yangzhou Jiangsu China; ^3^ State Key Laboratory of Food Science and Resources, Jiangnan University Wuxi Jiangsu China

**Keywords:** fried foods, heterocyclic amines, mycotoxin, QuEChERS, simultaneous extraction

## Abstract

Dietary exposure to both processing contaminants and natural toxins in fried foods poses a potential health risk, but analytical methods for their simultaneous monitoring are lacking. To address this gap, a rapid and efficient method based on QuEChERS extraction and UPLC–MS/MS detection was developed for the simultaneous determination of two β‐carboline heterocyclic amines (harman and norharman) and seven mycotoxins (fumonisins B1, B2, B3 and aflatoxins B1, B2, G1, G2). The optimized extraction utilized a 30:20:50 (v/v/v) acetonitrile/methanol/water mixture. The method was rigorously validated, showing high sensitivity (LODs: 0.021–0.232 ng/g; LOQs: 0.100–0.587 ng/g), accuracy (recoveries: 80.4%–109.3%), and reliability. When applied to real samples, the method not only confirmed its practicality across different matrices but also revealed a critical finding: the co‐occurrence of heterocyclic amines and mycotoxins, particularly in fried peanuts. This study provides an essential tool for integrated food safety surveillance and highlights the need for combined risk assessment of multiple contaminants.

## Graphical abstract

• A novel QuEChERS‐UPLC‐MS/MS method was successfully developed for the simultaneous determination of two β‐carboline heterocyclic amines and seven mycotoxins in fried foods.

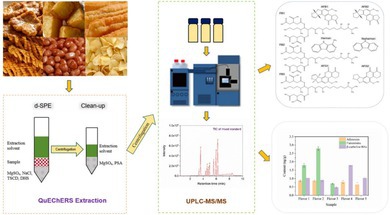

## 1 Introduction

Mycotoxins, toxic secondary metabolites produced by filamentous fungi, pose a significant global threat to food safety and public health (Xue et al. 2025). Their carcinogenic, mutagenic, teratogenic, and immunosuppressive properties can contaminate a vast array of agricultural commodities. Among the hundreds identified, aflatoxins and fumonisins are two of the most critical classes due to their frequent occurrence and severe toxicity (Chen et al. 2018). Aflatoxins, produced primarily by *Aspergillus flavus* and *Aspergillus parasiticus*, are commonly found in maize, peanuts, and tree nuts. This group mainly includes aflatoxin B1 (AFB1), B2 (AFB2), G1 (AFG1), and G2 (AFG2) (Kumar et al. 2008; Rajeev et al. 2010). AFB1 is the most prevalent and potent natural hepatocarcinogen known, classified by the International Agency for Research on Cancer (IARC) as a Group 1 carcinogen (IARC 2002). Its metabolism leads to DNA adduct formation, initiating mutagenic processes that can cause hepatocellular carcinoma (Kaale et al. 2021). Fumonisins, primarily produced by *Fusarium verticillioides* and *Fusarium proliferatum*, are common contaminants in maize and maize‐based products. Fumonisin B1 (FB1), B2 (FB2), and B3 (FB3) are the most abundant and toxicologically significant (Humpf and Voss 2004). FB1 disrupts sphingolipid metabolism, leading to toxic outcomes such as equine leukoencephalomalacia and porcine pulmonary edema (Humpf and Voss 2004). Epidemiological studies link dietary fumonisin exposure to an increased incidence of human esophageal cancer, leading IARC to classify FB1 as a Group 2B carcinogen (possibly carcinogenic to humans) (Ahangarkani et al. 2014). A major concern is the frequent co‐occurrence of these mycotoxins in grains, which can lead to additive or synergistic health risks. The primary human exposure route is through consuming contaminated food, making dietary intake a critical public health issue, with chronic low‐level exposure remaining a significant challenge.

Parallel to mycotoxins, Heterocyclic Amines (HAs) represent another class of hazardous compounds formed during high‐temperature food processing (Wu et al. 2025). These are generated in protein‐rich foods through the Maillard reaction between amino acids and reducing sugars, which also creates desirable flavors and colors (Gibis 2016). However, this process can yield undesirable HAs. Among over 30 mutagenic HAs identified, the β‐carbolines, norharman and harman, are particularly notable. While not directly mutagenic, they exhibit potent co‐mutagenic effects, enhancing the toxicity of other HAs (Diaodiao et al. 2019). Furthermore, they possess neuroactive properties, acting as monoamine oxidase inhibitors and interacting with neurotransmitter receptors. Abnormal levels have been linked to neurological disorders like Parkinson's disease and depression. Dietary sources include cooked meats, fish, and thermally processed plant‐based foods (Khan et al. 2017; Piechowska et al. 2019).

The convergence of these contaminant classes is particularly relevant in fried foods, which enjoy global popularity. The market for convenient, ready‐to‐eat fried snacks like potato chips continues to grow. The processing of these foods—slicing, washing, and deep‐frying at 160°C–180°C—creates an ideal environment for the Maillard reaction and HA formation. Tryptophan, naturally present or from flavoring additives, serves as a key precursor for β‐carboline HAs (Liu et al. 2021). Simultaneously, the raw materials (e.g., potatoes, grains for coatings) are susceptible to fungal infestation. Critically, mycotoxins like fumonisins and aflatoxins are thermally stable and persist through the frying process, leading to potential concurrent consumer exposure to both processing‐induced HAs and natural mycotoxins from a single food item. Despite this realistic co‐exposure scenario, research investigating the simultaneous presence and combined risk of these contaminants in complex fried snacks remains a significant niche.

Robust analytical methodologies are essential for accurate exposure assessment. Liquid chromatography coupled with tandem mass spectrometry (LC‐MS/MS) has become the gold standard for the sensitive and simultaneous determination of multiple analytes in complex matrices (Leite et al. 2020). However, the primary challenge lies in sample preparation. High‐fat, complex matrices like fried foods contain interfering substances (fats, oils, pigments) that can cause significant matrix effects, compromising accuracy and sensitivity. Traditional methods, such as solid‐phase extraction (SPE) for mycotoxins or liquid–liquid extraction for HAs, are often tailored to a single contaminant class (Romera et al. 2018; Zeng et al. 2017). They are typically time‐consuming, solvent‐intensive, and not readily adaptable for the simultaneous extraction of chemically diverse compounds like polar fumonisins, less polar aflatoxins, and basic β‐carboline HAs. Analyzing these groups separately doubles laboratory resources and fails to provide a holistic risk profile.

The need for efficient multi‐analyte methods has driven the adoption of well‐established techniques like QuEChERS (Quick, Easy, Cheap, Effective, Rugged, and Safe) (Wilkowska and Biziuk 2011). Originally developed for pesticide analysis, its principles—involving solvent extraction, salting‐out partitioning, and dispersive‐SPE clean‐up—have been successfully adapted for various analytes. Its advantages include simplicity, high throughput, low solvent consumption, and effective purification (Ferracane et al. 2021; Gonzalez‐Jartin et al. 2021). While QuEChERS applications for multi‐mycotoxin analysis or HAs individually are documented, developing a single, optimized method for the synchronous extraction of both mycotoxins (AFB1, AFB2, AFG1, AFG2, FB1, FB2, FB3) and β‐carboline HAs (norharman, harman) from high‐fat fried foods represents a significant analytical challenge and advancement.

Current methods are often singular in focus and cannot meet the demand for high‐throughput, rapid monitoring of multiple contaminant classes. Therefore, developing an efficient, synchronized method for extracting and determining key mycotoxins and β‐carboline HAs is crucial for comprehensive food safety surveillance. This study aimed to address this gap by developing, optimizing, and validating a novel QuEChERS‐based method for the synchronous extraction of two β‐carboline HAs and seven mycotoxins from various fried foods, with determination by UPLC‐MS/MS. The optimized method was applied to real‐world samples, including fried meat, fried potatoes, fried bread stick, fried twists, and fried peanuts. Furthermore, given their market dominance, a comparative analysis of different flavors of a commercial potato chip brand was conducted to provide data on the occurrence and potential co‐exposure of these toxicants in a popular snack.

## 2 Materials and Methods

### 2.1 Chemicals and Materials

Standards of harman and norharman (purity ≥ 98%) were acquired from Sigma‐Aldrich (Steinheim, Germany). Aflatoxin standards (AFB1, AFB2, AFG1, AFG2) and fumonisin standards (FB1, FB2, FB3) (purity ≥ 98%) were obtained from Santa Cruz Biotechnology Inc. (Dallas, TX, USA). HPLC‐grade methanol (MeOH), acetonitrile (ACN), and formic acid (FA) were supplied by Aladdin Reagent Co. Ltd. (Shanghai, China). Primary‐secondary amine (PSA), disodium hydrogen citrate sesquihydrate (DHS), anhydrous MgSO4, trisodium citrate dihydrate (TSCD), and NaCl were provided by Thermo Fisher Scientific (Waltham, MA, USA). Ultrapure water was produced using a Millipore Milli‐Q system (Bedford, MA, USA).

Stock standard solutions of aflatoxins and β‐carboline HAs were prepared in MeOH at 1 and 2 mg/mL, respectively. Fumonisin standards were dissolved in 50% (v/v) methanol/water to prepare 1 mg/mL stock solutions. Working solutions were prepared by serial dilution in methanol. All solutions were stored at −20°C in amber vials.

### 2.2 Sample Preparation

Fried food samples (fried meat, fried potatoes, fried bread stick, fried twists, and fried peanuts) and potato chips of different flavors (spicy, grilled pork, classic, roasted chicken wing, grilled squid) were purchased from a local supermarket in Shangqiu, China. Samples were homogenized using a blender and then ground into a fine powder with a mortar and pestle. The powdered samples were stored at 4°C until analysis.

### 2.3 Final QuEChERS Extraction and Clean‐Up Procedure

A 2.0 g (±0.1 g) aliquot of the homogenized sample was weighed into a 50 mL centrifuge tube. The sample was hydrated with 2 mL of ultrapure water and allowed to stand for 10 min. Subsequently, 10 mL of extraction solvent was added. The mixture was vortexed vigorously for 2 min. Then, a salt mixture containing 4 g anhydrous MgSO4, 1 g NaCl, 1 g TSCD, and 0.5 g DHS was added immediately (Lehotay 2007). The tube was shaken vigorously for 2 min and then centrifuged at 10,000 × g for 5 min at 4°C.

### 2.4 UPLC‐MS/MS Analysis

Chromatographic separation was performed on a Waters Acquity UPLC system (Milford, MA, USA) equipped with a Waters Acquity UPLC BEH C18 column (100 mm × 2.1 mm i.d., 1.7 μm) maintained at 35°C. The mobile phase consisted of (A) 0.1% formic acid in water and (B) methanol. The flow rate was 0.3 mL/min, and the injection volume was 2 μL. The gradient elution program was as follows: 0–1.0 min, 2% B; 1.0–4.0 min, 2%–20% B; 4.0–8.0 min, 20%–100% B; 8.0–9.0 min, 100% B; 9.0–9.1 min, 100%–2% B; and 9.1–10.0 min, 2% B for column re‐equilibration.

Mass spectrometric detection was carried out using a Waters QQQ‐MS spectrometer with an electrospray ionization (ESI) source in positive ion mode. The operational parameters were as follows: capillary voltage, 3.5 kV; source temperature, 100°C; desolvation temperature, 400°C; desolvation gas (N2) flow, 700 L/h; cone gas flow, 50 L/h. Data acquisition was performed in multiple reaction monitoring (MRM) mode. The optimized MRM transitions, cone voltages, and collision energies for each analyte are summarized in Table 1.

**TABLE 1:** Multiple reaction monitoring parameters for the identification and quantification of aflatoxins (AFB1, AFB2, AFG1, AFG2), fumonisins (FB1, FB2, FB3), and β‐carboline HAs (harman, norharman) by UPLC‐MS/MS.

	Precursor ion [M + H]^+^ (m/z)	Product ion (m/z)	Cone voltage (V)	Collision voltage (V)	Dwell time (s)
AFB1	313	285	30	20	0.2
AFB2	315	287	30	28	0.2
AFG1	329	243	30	25	0.2
AFG2	331	245	30	30	0.15
FB1	722.4	334	50	40	0.15
FB2	706.4	336	50	40	0.15
FB3	706.4	336	50	40	0.15
Harman	183	115	30	30	0.15
Norharman	169	115	30	30	0.15

### 2.5 Optimization of the QuEChERS Procedure

The extraction solvent and ultrasonic extraction time were optimized to achieve maximum recovery for all target analytes.

#### 2.5.1 Extraction Solvent

**FIGURE 1:**
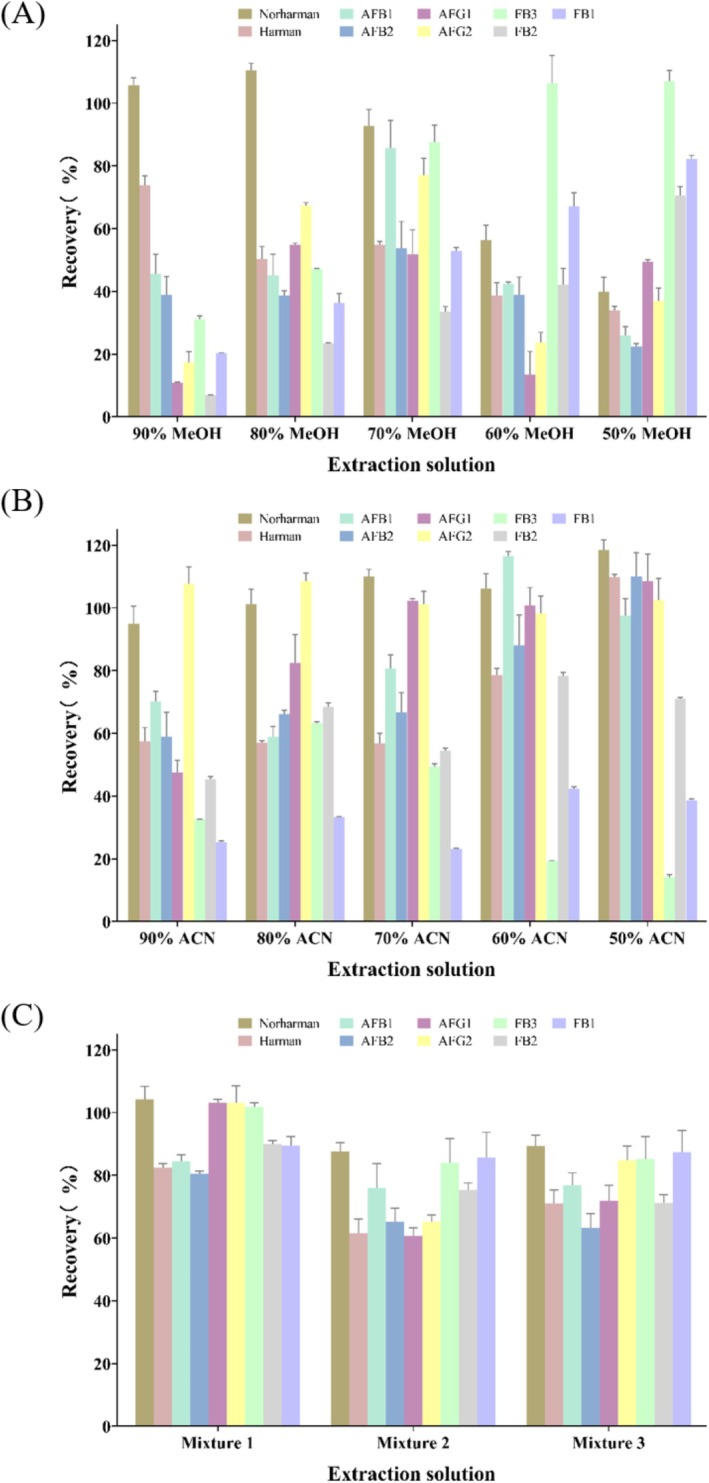
Selection of reagents during the simultaneous extraction of β‐carboline HAs (harman, norharman), fumonisins (FB_1_, FB_2_, FB_3_), and aflatoxins (AFB_1_, AFB_2_, AFG_1_, AFG_2_). Data are expressed as the mean ± SD (*n* = 3).

Several solvent systems were evaluated, including 50%–90% acetonitrile in water, 50%–90% methanol in water, and a mixture of acetonitrile, methanol, and water. Based on the overall recovery rates for all analytes, a mixture of 30% acetonitrile, 20% methanol, and 50% ultrapure water was selected as the optimal extraction solvent for all subsequent experiments (see results in Figure 1).

#### 2.5.2 Ultrasonic Extraction Time

**FIGURE 2:**
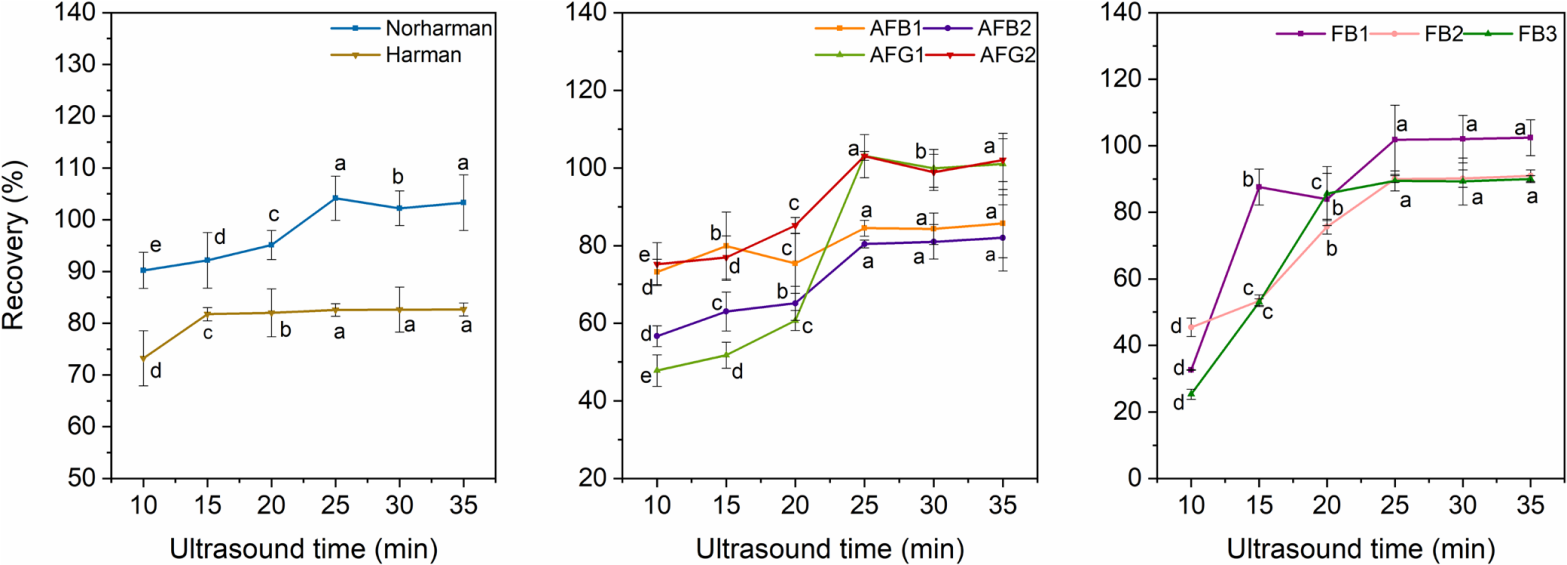
Optimization of ultrasonic extraction time of heterocyclic amines (harman, norharman), aflatoxins (AFB_1_, AFB_2_, AFG_1_, AFG_2_), and fumonisins (FB_1_, FB_2_, FB_3_). Data are expressed as the mean ± S.D. (*n* = 3). Different lowercase letters indicate that there are significant differences in the indexes measured by the same group of samples under different processing conditions (LSD, *p* < 0.05).

The influence of ultrasonic extraction time was investigated over a range of 10 to 35 min. An ultrasonic extraction time of 25 min was found to be optimal and was adopted for the final method (see results in Figure 2).

### 2.6 Method Validation

The developed method was validated in terms of linearity, limit of detection (LOD), limit of quantification (LOQ), precision, and recovery.

#### 2.6.1 Linearity

Calibration curves were constructed by analyzing nine different concentrations of mixed standard solutions in solvent and matrix‐matched blanks. The linear range for each compound was: Harman, 0.143–73 ng/mL; Norharman, 0.126–64.4 ng/mL; FB1, FB2, FB3, 0.25–500 ng/mL; AFB1, AFG1, 0.244–250 ng/mL; AFB2, AFG2, 0.489–250 ng/mL. Linearity was evaluated by the correlation coefficient (R^2^).

#### 2.6.2 LOD and LOQ

The instrumental LOD and LOQ were determined as the concentrations yielding signal‐to‐noise (S/N) ratios of 3 and 10, respectively. The method LOD and LOQ (in ng/g) were determined by spiking blank matrix samples (potato chips and fried twist) with decreasing levels of the analytes, followed by the complete extraction and subsequent UPLC‐MS/MS analysis. The S/N ratios of 3 and 10 from these processed matrix samples were used as the criteria for LOD and LOQ, respectively. These values therefore represent the sensitivity of the entire method within the relevant food matrices.

#### 2.6.3 Recovery and Precision

Recovery experiments were performed by spiking blank samples at low, medium, and high concentration levels (*n* = 5 for each level) prior to extraction. The recovery was calculated by comparing the measured concentration to the spiked concentration. The precision was expressed as the relative standard deviation (RSD%) of the recoveries.

### 2.7 Statistical Analysis

Data acquisition and processing were performed using Masslynx 4.1 software (Waters). All experiments were conducted in triplicate. Data are presented as mean ± standard deviation. Statistical analysis was performed using SPSS Statistics 9.0 software using one‐way ANOVA, and means were compared by the least‐significant difference (LSD) test at a significance level of *p* < 0.05. Graphical presentations were created using Origin 8.0 software.

## 3 Results and Discussion

### 3.1 Optimization of Extraction Solvent

The selection of an efficient extraction solvent is critical for the simultaneous recovery of analytes with diverse polarities, such as polar fumonisins, mid‐to‐non‐polar aflatoxins, and basic β‐carboline HAs. The extraction efficiency of aqueous acetonitrile and aqueous methanol at different concentrations was evaluated (Figure 1). Aqueous acetonitrile was effective for β‐carboline HAs and aflatoxins, with 50% acetonitrile yielding the highest recoveries for these compounds. However, the recovery of fumonisins remained low. Conversely, aqueous methanol improved the extraction of fumonisins, with 50% methanol showing the highest efficiency, but at the cost of lower recoveries for β‐carboline HAs and aflatoxins. This confirmed that neither pure aqueous acetonitrile nor aqueous methanol was suitable for the simultaneous extraction of all target analytes.

To achieve a balanced recovery for all analyte classes, mixed solvents of acetonitrile and methanol were investigated. A mixture of 30% acetonitrile, 20% methanol, and 50% ultrapure water (Mixture 1) provided superior and more balanced recoveries compared to other ratios (20:30:50 and 25:25:50). This can be attributed to the complementary solvation properties of the solvents: acetonitrile effectively precipitates proteins and extracts medium‐polarity compounds (aflatoxins, HAs) (Romera et al. 2018)., while methanol enhances the extraction of more polar fumonisins (Yan et al. 2017). The water content is crucial for swelling the matrix and facilitating the extraction of water‐soluble fumonisins. Therefore, Mixture 1 of 30% acetonitrile, 20% methanol, and 50% ultrapure water was selected as the optimal extraction solvent for all subsequent experiments.

### 3.2 Optimization of Extraction Time

Ultrasonication time is a key parameter that influences the mass transfer of analytes from the food matrix into the solvent. The effect of extraction time was studied from 10 to 35 min (Figure 2). The extraction efficiency for all analytes increased with sonication time up to 25 min. Prolonging the extraction beyond 25 min did not lead to a significant increase in the recovery for most analytes (e.g., harman, AFB1, FB1). Notably, the recoveries for norharman, AFG1, and AFG2 reached their maximum at 25 min (104.15%, 103.13%, and 103.06%). Therefore, an ultrasonication time of 25 min was adopted as the optimal condition, providing maximum extraction efficiency without unnecessarily prolonging the sample preparation process.

### 3.3 Method Validation

**FIGURE 3:**
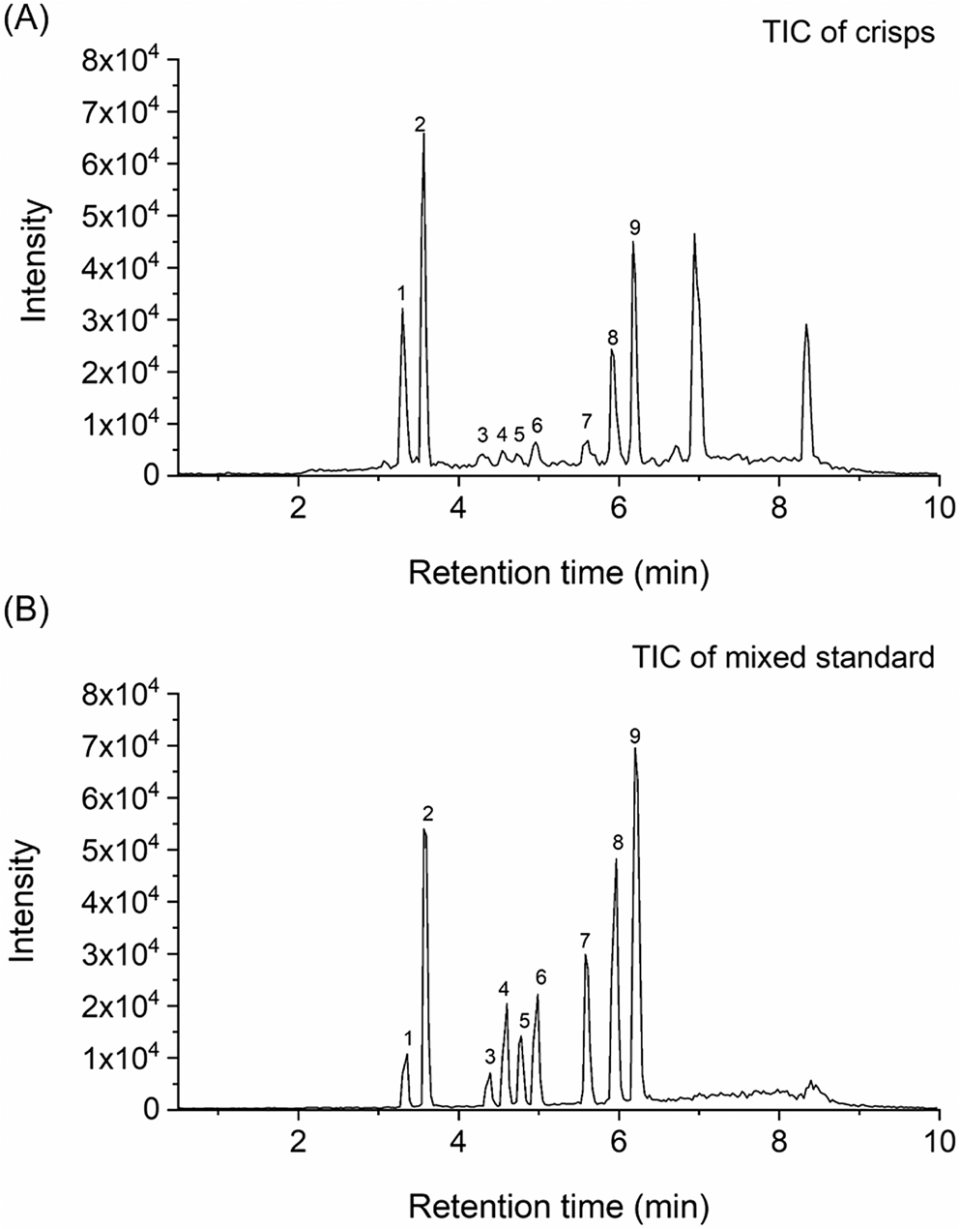
Total ion chromatograms (TICs) of β‐carboline HAs and seven mycotoxins in (A) a representative blank potato chip sample spiked with the standards at the medium level and (B) a mixed standard solution: 1. Norharman (*m/z* 169 → 115); 2. Harman (*m/z* 183 → 115); 3. AFG_2_ (*m/z* 331 → 245); 4. AFG_1_ (*m/z* 329 → 243); 5. AFB_2_ (*m/z* 315 → 287); 6. AFB_1_ (*m/z* 313 → 285); 7. FB_1_ (*m/z* 722.4 → 334); 8. FB_3_ (*m/z* 706.4 → 336); 9. FB_2_ (*m/z* 706.4 → 336).

The optimized QuEChERS method was validated for linearity, sensitivity, precision, and accuracy in potato chip and fried food matrices. Figure 3 shows total ion chromatograms (TICs) of β‐carboline HAs and seven mycotoxins in (A) a mixed standard solution and (B) a representative blank potato chip sample spiked with the standards at the medium level.

Linearity, LOD, and LOQ: The method demonstrated good linearity over a wide concentration range for all analytes in both matrix‐matched calibrations, with correlation coefficients (R^2^) greater than 0.9990 for most compounds (Table 2). The LODs and LOQs ranged from 0.021 to 0.232 ng/g and 0.100 to 0.587 ng/g, respectively (Table 2). These values are sufficiently low to monitor the target analytes at the levels expected in commercial samples and are comparable to or better than those reported in other studies for these compounds (Diaodiao et al. 2020; Lago et al. 2021).

**TABLE 2:** Working linear ranges, determination coefficients (R^2^), limits of detection (LODs), limits of quantification (LOQs), precisions (% RSD), and recoveries for the quantification of HAs (harman, norharman) and mycotoxins (FB_1_, FB_2_, FB_3_, AFB_1_, AFB_2_, AFG_1_, AFG_2_) in both fried twist and potato chips combined with QuEChERS.

Matrix	Target	Linear range (ng/mL)	Coefficients (R2)	LOD (ng/g)	LOQ (ng/g)	RSD (%)	Recovery (%)
Fried twist	Harman	0.015–73.0	0.9996	0.021	0.114	4.26	96.26
	Norharman	0.025–64.4	0.9994	0.055	0.100	3.64	99.00
	FB1	0.153–1000	0.9993	0.141	0.510	6.90	101.79
	FB2	0.076–1500	0.9990	0.230	0.490	7.71	92.99
	FB3	0.076–1000	0.9987	0.161	0.305	1.39	93.67
	AFB1	0.195–125	0.9998	0.175	0.443	2.81	88.54
	AFB2	0.097–250	0.9997	0.139	0.457	5.80	109.28
	AFG1	0.172–125	0.9990	0.119	0.479	5.96	99.92
	AFG2	0.186–250	0.9994	0.232	0.454	8.72	105.68
Potato chips	Harman	0.015–73.0	0.9993	0.028	0.198	5.13	82.54
	Norharman	0.051–64.4	0.9990	0.073	0.266	4.53	104.15
	FB1	0.153–1000	0.9992	0.164	0.555	3.00	89.45
	FB2	0.076–1500	0.9985	0.138	0.522	1.03	90.03
	FB3	0.076–1000	0.9971	0.139	0.363	1.39	101.8
	AFB1	0.195–125	0.9992	0.107	0.331	9.39	84.51
	AFB2	0.097–250	0.9990	0.178	0.393	7.09	80.43
	AFG1	0.172–125	0.9998	0.126	0.587	2.96	103.13
	AFG2	0.186–250	0.9998	0.158	0.449	6.72	103.06

Precision and Accuracy: The accuracy and precision of the method were assessed through recovery experiments. As shown in Table 2, the recoveries for all across different spiked matrices (potato chips and fried twist) ranged from 80.4% to 109.3%, with relative standard deviations (RSDs) below 10%. Similarly, satisfactory recoveries and RSDs were obtained in other fried food matrices (data for selected foods shown). These results confirm that the method is both accurate and precise.

The obtained validation parameters are fully fit‐for‐purpose. The recoveries and precision are comparable to those in literature for the individual analysis of these contaminant classes. For instance, recoveries for mycotoxins in complex matrices typically range from 63% to 113% (Garcia‐Moraleja et al. 2015; Rausch et al. 2020; Reichert et al. 2018), while recoveries for β‐carboline HAs are often above 80% (Chiang et al. 2022; Diaodiao et al. 2019; Yan et al. 2017). The key achievement of this work is the successful unification of these analyses into a single, efficient method that delivers performance metrics on par with, or superior to, those of dedicated single‐class methods.

### 3.4 Analysis of HAs and Mycotoxins in Fried Foods

For each of the five food categories (fried meat, fried potatoes, fried bread stick, fried twists, fried peanuts), three independent commercial samples were purchased and analyzed individually (*n* = 3). The validated method was applied to analyze a variety of commercially available fried foods (Table 3). The results revealed distinct contamination profiles. The total content of β‐carboline HAs was highest in fried peanuts (323.020 ng/g), followed by fried bread stick (50.870 ng/g) and fried twist (32.910 ng/g). The high levels in peanuts are likely due to their high protein and free amino acid (e.g., tryptophan) content, coupled with the thermal processing conditions that favor HA formation (Khan et al. 2017).

**TABLE 3:** The content of HAs (harman, norharman) and mycotoxins (FB_1_, FB_2_, FB_3_, AFB_1_, AFB_2_, AFG_1_, AFG_2_) in fried food (ng/g).

	Fried meat	Fried potato	Fried bread stick	Fried twist	Fried peanut
Harman	6.741 ± 0.090	3.747 ± 0.050	4.280 ± 0.100	13.351 ± 0.050	187.630 ± 0.100
Norharman	7.873 ± 0.110	13.436 ± 0.100	46.590 ± 0.160	19.557 ± 0.090	135.390 ± 0.560
HAs	14.614 ± 0.200	17.183 ± 0.150	50.870 ± 0.260	32.908 ± 0.140	323.020 ± 0.660
FB1	nd	0.940 ± 0.070	69.541 ± 2.310	nd	nd
FB2	nd	0.630 ± 0.050	nd	nd	nd
FB3	nd	0.661 ± 0.020	nd	nd	nd
FBs	nd	2.231 ± 0.140	69.541 ± 2.310	nd	nd
AFB1	nd	nd	nd	nd	nd
AFB2	nd	nd	nd	nd	17.654 ± 0.140
AFG1	nd	nd	nd	nd	nd
AFG2	nd	nd	nd	nd	3.662 ± 0.030
AFs	nd	nd	nd	nd	21.316 ± 0.170

Abreviations: nd, means lower than the limit of detection; nq, means lower than the limit of quantification.

Regarding mycotoxins, fumonisins (FBs) were detected exclusively in fried potato (total FBs: 2.231 ng/g) and fried bread stick (FB1: 69.541 ng/g), suggesting potential raw material contamination, possibly from maize‐based ingredients (Murtaza et al. 2025). Aflatoxins were only found in fried peanuts, with AFB2 (17.654 ng/g) and AFG2 (3.662 ng/g) being detected, yielding a total AFs content of 21.316 ng/g. The co‐occurrence of high levels of processing contaminants (HAs) and natural contaminants (mycotoxins) in a single food product (fried peanuts), potentially leading to additive or synergistic health risks, as both groups of compounds are known for their carcinogenic potential. The absence of AFB1 and AFG1 in all samples, along with the low or non‐detectable levels of other mycotoxins in most products, highlights the specificity of contamination. All detected mycotoxin levels were below the maximum limits set by the European Commission, but the simultaneous presence of multiple toxicants warrants attention from a cumulative risk assessment perspective. These findings underscore the importance of simultaneous monitoring of multiple processing contaminants and suggest that mitigating strategies, such as optimizing frying conditions and stringent raw material screening, should be implemented to enhance the safety of fried food products, especially those derived from peanuts and certain cereal matrices.

### 3.5 Analysis of HAs and Mycotoxins in Potato Chips

For each of the five potato chip flavors, three independent packages were purchased and analyzed individually (*n* = 3). All analyses were performed in triplicate. As shown in Table 4, the highest levels of aflatoxins (0.869 ± 0.080 ng/g), fumonisins (2.792 ± 0.104 ng/g), and β‐carboline HAs (1.796 ± 0.045 ng/g) were detected in the grilled pork flavor and roasted chicken wing flavor, respectively. Aflatoxins and β‐carboline HAs were the least abundant in the classic flavor, but fumonisins did not reach the LOQ in roasted chicken wing flavor and grilled squid flavor. This variation could be attributed to different seasoning blends and their raw material quality (Li et al. 2023). According to the European Commission, the maximum level of aflatoxins including AFB1, AFB2, AFG1, and AFG2 in all cereals and cereal products was 4.0 μg/kg and the maximum level of fumonisins including FB1 and FB2 in maize food for direct human consumption was 400 μg/kg (EU 2023). It has been reported that aflatoxins in potatoes can reach 2 to 9 μg/kg (Zöngür 2020). The content of aflatoxins in sweet potato chips varied from 10.49 to 75.12 μg/kg (Amri and Lenoi 2016).

**TABLE 4:** The content of aflatoxins (AFB1, AFB2, AFG1, AFG2), fumonisins (FB1, FB2, FB3), and β‐carboline HAs (harman, norharman) in potato chips with different flavors (ng/g).

Potato chips	Pure spicy flavor	Grilled pork flavor	Classic flavor	Roasted chicken wing flavor	Grilled squid flavor
Harman	0.380 ± 0.007^b^	0.339 ± 0.047^c^	0.193 ± 0.018^d^	0.440 ± 0.028^a^	0.196 ± 0.035^d^
Norharman	0.642 ± 0.056^c^	0.570 ± 0.026^d^	0.276 ± 0.014^e^	1.356 ± 0.017^a^	0.826 ± 0.020^b^
HAs	1.022 ± 0.063^b^	0.909 ± 0.073^c^	0.469 ± 0.032^d^	1.796 ± 0.045^a^	1.022 ± 0.055^b^
FB1	0.606 ± 0.043^b^	1.638 ± 0.008^a^	0.691 ± 0.022^b^	nd	nq
FB2	0.657 ± 0.042^a^	0.683 ± 0.050^a^	nd	nq	nq
FB3	0.530 ± 0.027^a^	0.471 ± 0.046^a^	nd	nq	nq
Fumonisins	1.793 ± 0.112^b^	2.792 ± 0.104^a^	0.691 ± 0.022^c^	nq	nq
AFB1	nq	nq	nd	nq	nq
AFB2	nq	nq	nq	nq	nq
AFG1	nq	nq	nq	nq	nd
AFG2	0.865 ± 0.049^a^	0.869 ± 0.080^a^	nq	0.798 ± 0.084^b^	0.634 ± 0.127^c^
Aflatoxins	0.865 ± 0.049^a^	0.869 ± 0.080^a^	nq	0.798 ± 0.084^b^	0.634 ± 0.127^c^

*Note:* Comparisons are made within the same row.

Abbreviations: nd, means lower than the limit of detection; nq, means lower than the limit of quantification.

^a^ Provides statistical significance at *p* value = 0.05.

^b^ Provides statistical significance at *p* value = 0.05.

^c^ Provides statistical significance at *p* value = 0.05.

^d^ Provides statistical significance at *p* value = 0.05.

^e^ Provides statistical significance at *p* value = 0.05.

More specifically, AFB1, AFB2, and AFG1 could be detected but not quantified without reaching the LOQ in potato chips with different flavors. AFG2, FB1, FB2, FB3, harman, and norharman were quantified in five flavors of potato chips. The content of AFG2 was below LOQ and could not be quantified in classic flavor chips. Moreover, the content of AFG2 was highest in grilled pork flavor potato chips (0.869 ± 0.080 ng/g), and the highest content of harman and norharman was detected in roasted chicken wing flavor potato chips (Table 4). Potato chips are very popular, and consumers tend to eat a large amount. The quantified levels of all contaminants were, again, well below EU regulatory limits. However, given the high consumption volume of potato chips, especially by children, monitoring these contaminants remains important for assessing long‐term, low‐level dietary exposure. In understanding this wide range of consumption, determining harmful substances in potato chips is very important.

## 4 Conclusion

In this study, a novel QuEChERS‐based method was developed for the simultaneous determination of β‐carboline HAs and seven mycotoxins in fried foods. The optimized method, utilizing a 30:20:50 acetonitrile‐methanol–water mixture as the extraction solvent with a 25‐min ultrasonication step, proved to be rapid, sensitive, and reliable, demonstrated excellent performance with satisfactory validation parameters. It provided satisfactory recoveries (80.4%–109.3%), precision (RSDs < 10%), and high sensitivity (LODs in the low ng/g range) for all nine target analytes. The total analysis time of only 10 min per sample underscores the high‐throughput capability of this approach. Its application to commercial samples not only demonstrated robustness across complex matrices but also uncovered critical co‐occurrence patterns, most notably the simultaneous presence of high levels of β‐carboline HAs and aflatoxins in fried peanuts.

These findings underscore the significance of moving beyond single‐contaminant monitoring. The presented method provides a practical tool for comprehensive risk assessment, enabling a more holistic approach to ensuring the safety of thermally processed snacks. Future research should utilize this method for larger‐scale surveillance and to explore the toxicological implications of the co‐exposure phenomena revealed in this study.

## Author Contributions

Diaodiao Yang: conceptualization, methodology, data curation, writing – original draft preparation, funding acquisition. Jianwen Liu: conceptualization, methodology, data curation, writing – original draft preparation, funding acquisition. Yang Song: writing – reviewing and editing. Yajing Ma: writing – reviewing and editing. Hongkang Zhu: writing – reviewing and editing. Qingfeng Zhou: writing – reviewing and editing, formal analysis, project administration. Maomao Zeng: writing – reviewing and editing, project administration, funding acquisition.

## Funding

This work was supported by Henan Provincial Science and Technology Research Project, 252102321124. Scientific Research Start‐up Fund Project of Shangqiu Normal University, SQNUQDF2504. National Natural Science Foundation of China, 32272430. Natural Science Foundation of Jiangsu Province, BK20250939. The “Lüyangjinfeng” Talent Support Program of Yangzhou City.

## Conflicts of Interest

The authors declare no conflicts of interest.

## Data Availability

The data that support the findings of this study are available from the corresponding author upon reasonable request.
